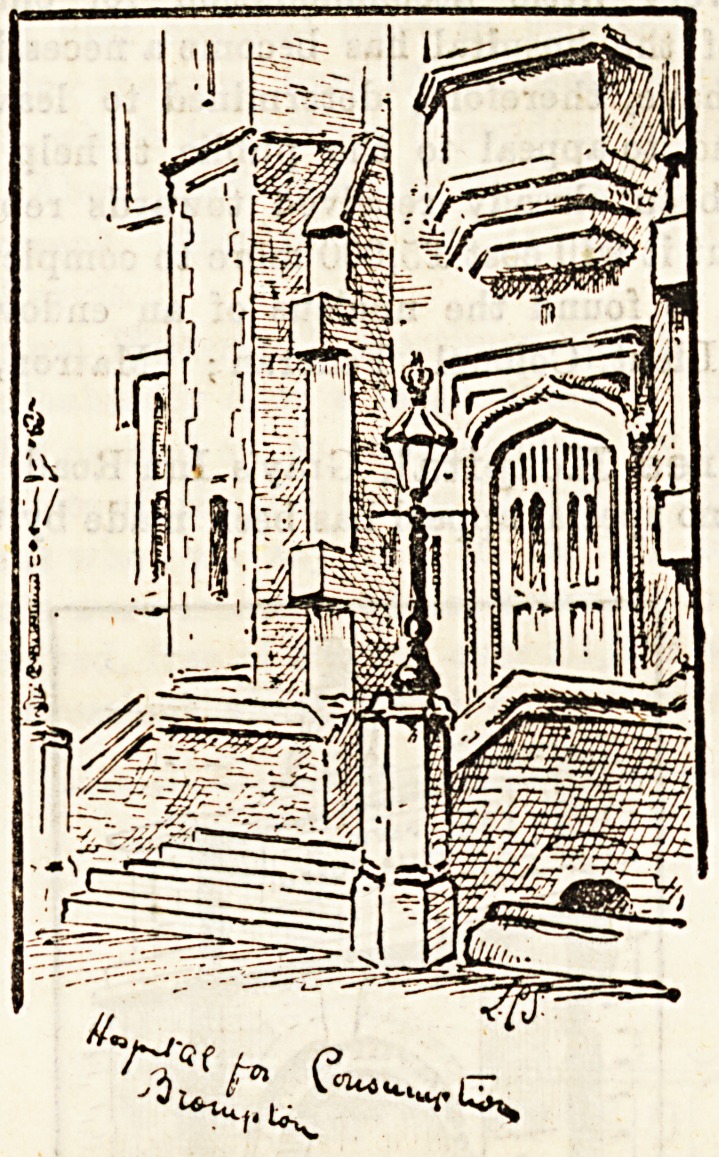# Consumption

**Published:** 1892-12-24

**Authors:** 


### SPECIAL HOSPITALS.

### CONSUMPTION.

**Brompton Hospital for Consumption and Diseases of the Chest,** S.W.—This hospital is well known as one where the patients are well cared for, and every effort is made to brighten their clouded lives. The maintenance of the new extension building entails an extra expenditure of several thousand pounds a year, and funds are urgently needed to meet this outlay. Secretary, Mr. H. Dobbin.

**City of London Hospital for Diseases of the Chest,** Victoria Park, E.—The average expenditure is about £10,000, towards which only £370 of the income is assured. Altogether about 1,000 in-patients and 15,000 out-patients are treated yearly. Secretary, Mr. T. Storrar-Smith; Matron, Miss H. G. Hetherington.

**North London Hospital for Consumption,** Mount Vernon, Hampstead, N.W.—This institution was thoroughly reorganised and remodelled some years ago, and has now become one of the most useful of its class. Though not so large as the above, yet 400 in, and 3,000 out-patients are admitted each year. Secretary, Mr. Lionel F. Hill, M.A.; Matron, Miss Blaxland.

**Royal Hospital for Diseases of the Chest,** City Road, E.C.—Five hundred and twenty-three in-patients and 8,662 out-patients were relieved in 1891. Seven-eighths of the patients are of the hard-working and wage-earning class. Two out of the four wards in the wing opened in 1886 are at the present time closed for want of funds. Secretary, Mr. John Harrold; Matron, Miss M. L. Smith.

## Figures and Tables

**Figure f1:**